# Sustainability in Health care by allocating resources effectively (SHARE) 1: introducing a series of papers reporting an investigation of disinvestment in a local healthcare setting

**DOI:** 10.1186/s12913-017-2210-7

**Published:** 2017-05-04

**Authors:** Claire Harris, Sally Green, Wayne Ramsey, Kelly Allen, Richard King

**Affiliations:** 1 School of Public Health and Preventive Medicine, Monash University, Victoria, Australia; 2 Centre for Clinical Effectiveness, Monash Health, Victoria, Australia; 3 Medical Services and Quality, Monash Health, Victoria, Australia; 4 Medicine Program, Monash Health, Victoria, Australia

**Keywords:** Disinvestment, Decommission, De-adopt, De-list, De-implement, Health technology, TCP, Resource allocation, Decision-making, Implementation

## Abstract

This is the first in a series of papers reporting Sustainability in Health care by Allocating Resources Effectively (SHARE). The SHARE Program is an investigation of concepts, opportunities, methods and implications for evidence-based investment and disinvestment in health technologies and clinical practices in a local healthcare setting. The papers in this series are targeted at clinicians, managers, policy makers, health service researchers and implementation scientists working in this context. This paper presents an overview of the organisation-wide, systematic, integrated, evidence-based approach taken by one Australian healthcare network and provides an introduction and guide to the suite of papers reporting the experiences and outcomes.

## Background

The primary focus of health care should be on optimising patient outcomes, but without due consideration of value for money healthcare systems will not be sustainable [1, 2]. There are many challenges to the sustainability of healthcare services. Ageing populations and the increasing prevalence of chronic diseases, the proliferation and high costs of new health technologies, duplication and gaps in service delivery from poorly coordinated care, ineffective practices, systemic waste and external economic pressures all threaten the ability to maintain health services at acceptable standards [3–10].

In the first decade of this century healthcare expenditure rose steadily, in total and as a percentage of gross domestic product (GDP) [11]. The average for countries in the Organisation for Economic Co-operation and Development (OECD) rose from 8.2% GDP in 2001 to 9.3% 10 years later [11]. Advances in technology are considered to be a major driver of increased costs [12–14]. In 2011 the global health technology market was valued at US$325 billion with an annual growth rate of 7% [15]. It has been estimated that health technologies account for 25–48% of health spending growth [16, 17]. The growth is not just due to adoption of new technology but also to rapidly increasing use of existing technology [12].

However, since 2010 the growth in global health care expenditure has plateaued and many countries have reduced public spending on health [11]. This has directed attention towards opportunities to save money, reduce waste and maximise outcomes from existing resources.

Many healthcare interventions reduce costs by improving timely access to treatment, facilitating earlier diagnosis, enhancing patient outcomes, decreasing hospital stays or minimising side effects, and provide value by increasing quality or length of life. Unfortunately it is also true that many interventions do not provide these benefits and the outcomes of many others are unknown. It has been estimated that “*a third of medical practices are effective or likely to be effective; 15% are harmful, unlikely to be beneficial, or a trade-off between benefits and harms; and 50% are of unknown effectiveness*” [18]. The cost-effectiveness is even less well known [14].

It is now customary to thoroughly appraise new technologies and procedures before introducing them into widespread use. Health Technology Assessment (HTA) involves systematic evaluation of safety, effectiveness and cost-effectiveness and often includes broader social and ethical impacts. However many practices in current use were not subjected to this rigorous evaluation prior to their introduction and would not meet contemporary standards [19]. In Australia, only 3% of all items on the Medicare Benefits Schedule have been formally assessed against evidence of safety, effectiveness and cost-effectiveness [20]. Reviews of the international literature have found that many interventions were implemented based on early evidence and the initial promising findings were reversed in subsequent studies [18, 21, 22]. Even practices that have clearly demonstrated benefits may be applied inappropriately or incorrectly [23–25]. These issues can be seen as shortcomings, or viewed more constructively as opportunities to improve patient outcomes, optimise use of resources and possibly save money by removing or restricting practices that are unsafe or of little value.

Health authorities, hospitals and other health facilities have always moved resources from one area to another to achieve better clinical or corporate outcomes. Previously, decisions to restrict or reallocate resources were generally reactive, undertaken in response to established or emerging problems, and the processes and assumptions underpinning them were frequently implicit and opaque. However in the past two decades proactive, explicit and transparent methods have been sought to address rising health costs and the need to meet continuing advances in expensive technologies. Debate and research have focused on practices that offer little or no benefit, or where a better alternative is available, and the concept of disinvestment has emerged.

The early research in this area concentrated on projects guided by health economic principles to disinvest specific technologies or clinical practices (TCPs) in a local setting, while the broader discussion focused on central policy-making and the role of national agencies to inform decision-making [26–28]. Although both play a vital role, there are limitations to these approaches. Individual projects can potentially be instigated and implemented independently of organisational goals, priorities, decision-making systems and communication processes. They may be driven by *ad hoc* decisions or individual champions and be undertaken in isolation from other local initiatives resulting in lack of coordination, duplication, inconsistent messages and change fatigue [29]. National recommendations cannot take into account local factors such as population needs, organisational priorities, budgets, capacity or capability; hence many crucial decisions about the use of TCPs have to be made at regional and institutional levels.

Although the research and debate has broadened considerably, a number of significant gaps remain. There is little evidence to guide healthcare networks or individual facilities in how they might take a systematic organisation-wide approach to disinvestment [26, 30–34]. There is also a lack of information about the factors that influence resource allocation, the processes involved in implementation of disinvestment decisions, and the perspectives and experiences of healthcare staff undertaking disinvestment [29, 34–38]. It has been proposed that in-depth research using longitudinal approaches from inception to implementation of disinvestment decisions at the health service level is needed to close these gaps and contribute to both the theory and practice of disinvestment [29, 35, 36, 39, 40].

The ‘Sustainability in Health care by Allocating Resources Effectively’ (SHARE) Program was the approach taken by one Australian health service to address these issues at the local level. The resulting suite of papers may contribute in part to filling these gaps [41–50].

## Aims

The aim of the SHARE Program was to establish organisation-wide, systematic, integrated, transparent, evidence-based systems and processes for decision-making about disinvestment in the context of resource allocation at Monash Health.

The aims of the SHARE series of publications are 1) to present the experiences and outcomes of the SHARE Program, 2) to review and discuss the current literature from the perspective of the local healthcare setting and 3) to propose frameworks and methods to inform future work in this area.

The aims of this paper are 1) to provide an overview of the SHARE Program, 2) to orient readers in how to find information and resources in this suite of publications, and 3) to discuss the contribution of the outputs of the program to policy, practice and research in disinvestment. The outcomes of SHARE are discussed in the final paper [50].

## The SHARE program

### Context

Monash Health (previously Southern Health), in the south east of Melbourne, Australia, is the largest health service network in the state of Victoria. It delivers primary, secondary, tertiary and quaternary services across more than 40 sites including six acute hospitals, subacute and rehabilitation services, mental health and community health services, and residential aged care [51]. Services are provided across the lifespan from conception and antenatal care through to care of the elderly; and all clinical specialties are offered.

Australian public hospitals operate under a state-allocated activity-based fixed-budget model of financing [52]. Staff are salaried and services are provided free of charge.

Monash Health established the first Technology/Clinical Practice Committee in Victoria to assess new TCPs prior to their introduction within the health service [53]. Australia has robust evidence-based processes for assessment at national level, however they do not address all the needs of health service decision-makers [53] and, as noted above, there are many reasons why decisions are required at local level. Although early leaders in this area, the Monash Health committee acknowledged that there were opportunities for improvement in their processes and undertook a project to identify and implement international best practice [53].

To build on this work, Monash Health leaders sought to explore the potential for a similar systematic organisation-wide approach to disinvestment of established practices that were unsafe, ineffective or inefficient or where better alternatives were available; and the SHARE Program was born.

The SHARE Program was undertaken by the Centre for Clinical Effectiveness (CCE), an Evidence Based Practice (EBP) Hospital Support Unit within Monash Health [54, 55]. Its role is to enable clinicians, managers and policy makers to use the best available evidence to improve healthcare decision-making. CCE facilitates knowledge translation by providing expertise, education and support in evidence synthesis and implementation and evaluation of evidence-based change; and delivering programs and projects underpinned by EBP. Consultants in health program evaluation and health economics were engaged to provide additional expertise to the SHARE project team.

The program was governed by a Steering Committee comprised of three Executive Directors (Medical, Nursing and Support Services), Clinical Program Directors (Medical, Nursing, Allied Health, Pharmacy and Diagnostic Services), Chairs of key committees (Technology/Clinical Practice, Therapeutics, Human Research and Ethics, and Clinical Ethics), representatives from relevant support services (Information Services, Procurement, Biomedical Engineering and Research Services), Legal counsel and two Consumer representatives.

### Design

#### Case study

The SHARE papers present a case study of disinvestment in the local healthcare setting. This approach seeks to address the limited understanding of resource allocation processes in health services, particularly regarding disinvestment [35, 36], and the lack of detailed reporting of implementation of change in the literature [56, 57]. Case studies allow in-depth, multi-faceted explorations of complex issues in their real-life settings [58] and facilitate development of theory and interventions [59]. The case study approach enables examination of the complex behaviours of, and relationships among, actors and agencies; and how those relationships influence change [60]. All three case study approaches are used: description, exploration and explanation [61].

#### Framework for design and evaluation of complex interventions

**Fig. 1:**
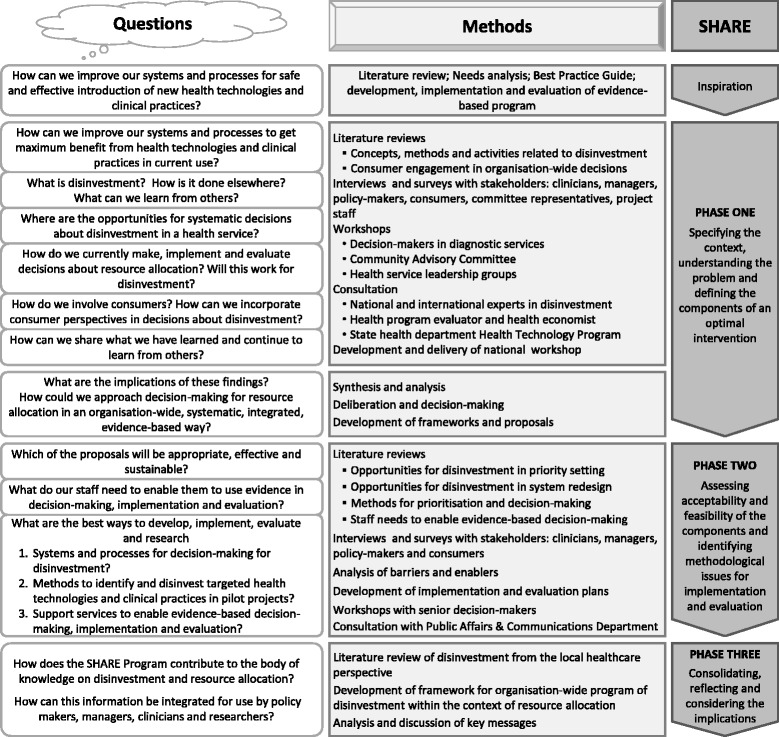
Overview of the SHARE Program

When a review of the literature found no specific information to guide development of an organisation-wide approach at the local health service level, a two-phased program based on the UK Medical Research Council framework for design and evaluation of complex interventions was proposed (Fig. 1) [62]. Phase One includes specifying the context, understanding the problem and defining the components of an optimal intervention. Phase Two is a series of exploratory trials assessing acceptability and feasibility of the components and identifying methodological issues for implementation and evaluation.

The questions outlined in Fig. 1 reflect the information needs of Monash Health decision-makers as they emerged in the respective phases of the SHARE process. The methods used to address these questions are noted alongside.

#### Model for evidence-based change

The SHARE Program was undertaken using the SEAchange model for Sustainable, Effective and Appropriate change in health services [63]. The model involves four steps: identifying the need for change, developing a proposal to meet the need, implementing the proposal and evaluating the extent and impact of the change. Each step is underpinned by the principles of evidence-based practice to ensure that the best available evidence from research and local data, the experience and expertise of health service staff and the values and perspectives of consumers are taken into account. Sustainability, avoidance of duplication and integration of new processes within existing systems are considered at each step. An action research component enables continuous investigation of the change process to improve the current project and inform future work.

**Fig. 2:**
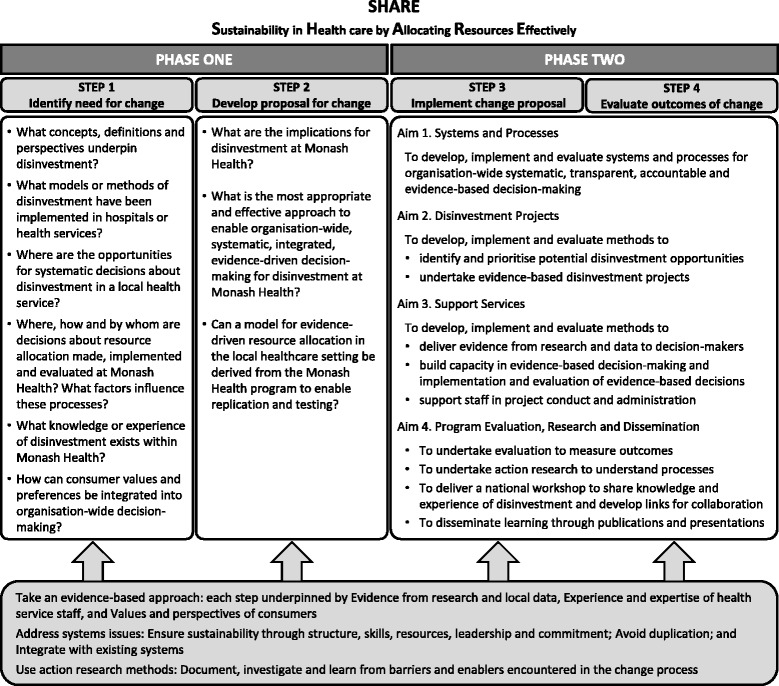
SEAchange model for evidence-based change adapted for SHARE (with permission from Harris et al [63])

The principles of this model were applied to the whole SHARE Program and to each individual project. In the overall SHARE Program, Steps 1 and 2 of the model map to Phase One and Steps 3 and 4 correspond to Phase Two (Fig. 2). The questions asked by decision-makers have been reframed as the research questions addressed in the SHARE papers.

#### Frameworks for evaluation and explication

Evaluation frameworks and plans were created for the SHARE Program as a whole [64] and for individual projects.

**Fig. 3:**
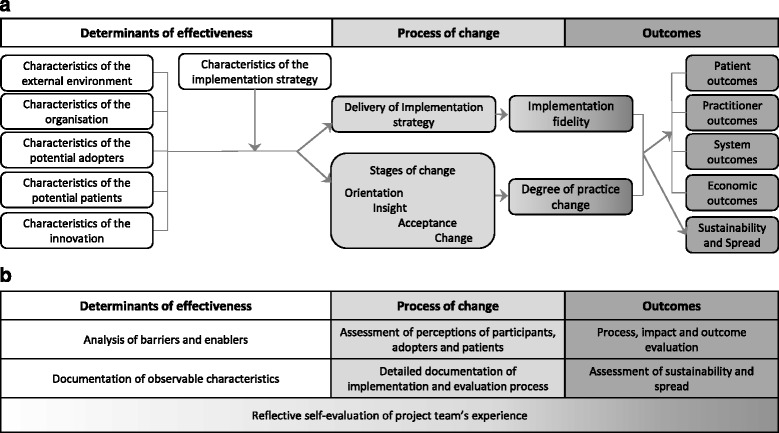
Framework for evaluation and explication of implementation of an evidence-based innovation (adapted with permission from Harris et al [65]) **a** Components, **b** Evaluation and research activities for SHARE Program and pilot projects

**Fig. 4:**
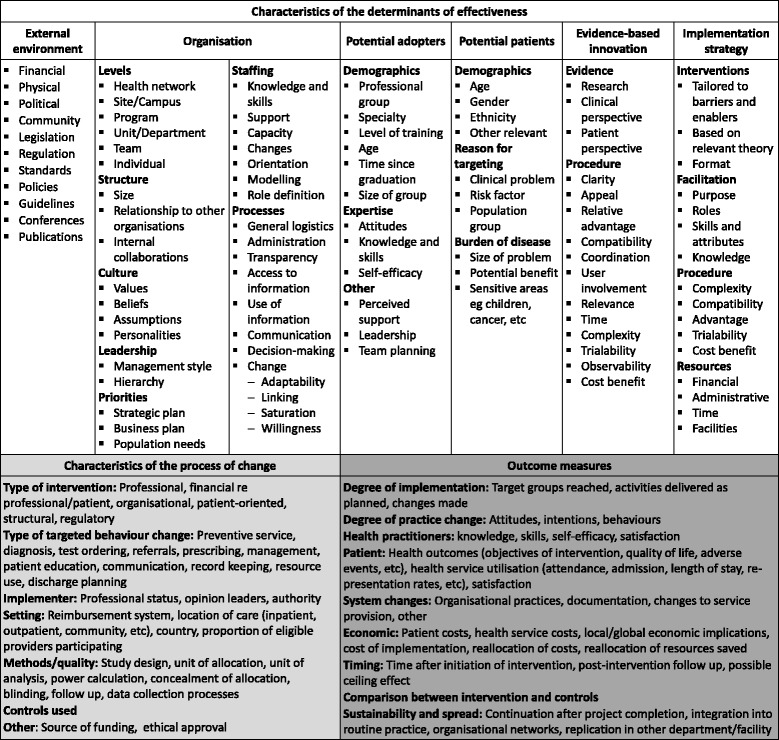
Taxonomy for evaluation and explication of implementation of an evidence-based innovation (adapted with permission from Harris et al [65])

A framework and associated taxonomy for evaluation and explication of implementation of an evidence-based innovation were adapted for use in SHARE activities (Figs. 3a and 4) [65]. Evaluation and research activities were mapped to the corresponding components of the framework (Fig. 3b).

### Activities and publications

The activities in Phase One focused on understanding disinvestment from the local health service perspective and identifying potential mechanisms for a systematic organisation-wide approach; discovering where, how and by whom decisions are made, implemented and evaluated at Monash Health; and exploring opportunities and methods for consumer engagement in this process. These are reported in Papers 2, 3 and 4 respectively [41–43]. A national workshop was conducted to share knowledge about disinvestment from three perspectives: health policy researchers, health economists and health service decision-makers. A report containing all findings and presentation materials is available [66, 67].

Following synthesis and analysis of the findings from these investigations and consideration of the implications that emerged, a plan for a multi-faceted disinvestment program was established. This is presented as a model for a systematic approach to evidence-based resource allocation in a local health service in Paper 5 [44].

Phase Two involved development, implementation and evaluation of the activities proposed in the model to determine which were sustainable, effective and appropriate at Monash Health. These projects are reported in Papers 6, 7 and 8 [45–47].

After completion of Phase Two a review of the disinvestment literature from the perspective of the local health service was undertaken and the findings were integrated with the experiences and outcomes of the SHARE Program in Paper 9 [48]. Although there is little practical guidance in the literature, there are clear and consistent messages regarding principles for decision-making, settings and opportunities to identify disinvestment targets, steps in the disinvestment process, methods and tools, and barriers and enablers. This information was drawn together into an organisation-wide framework for disinvestment in the local healthcare setting in Paper 10 [49].

Paper 11 summarises the outcomes of the SHARE Program, discusses the contribution of SHARE to the knowledge and understanding of disinvestment in the local setting, and considers the implications for research, policy and practice [50].

To aid readers in navigation of this series, the research questions addressed in each paper are listed in Table 1.

**Table 1:** Research questions and outputs

Research questions	Outputs
SHARE 2: Identifying opportunities for disinvestment in a local healthcare setting	
▪ What concepts, definitions and perspectives underpin disinvestment? ▪ What models or methods of disinvestment have been implemented in hospitals or health services? ▪ Where are the opportunities for systematic decisions about disinvestment in a local health service network?	▪ Framework and detailed discussion of potential settings and methods for disinvestment in the local healthcare context ▪ Summary of issues to consider in development of an organisational program for disinvestment ▪ Interview protocol for ascertaining local implications for disinvestment
SHARE 3: Examining how resource allocation decisions are made, implemented and evaluated in a local healthcare setting	
▪ Where, how and by whom are decisions about resource allocation made, implemented and evaluated at Monash Health? ▪ What factors influence these processes? ▪ What knowledge or experience of disinvestment exists within Monash Health?	▪ Framework of eight components in the research allocation process, the elements of structure and practice for each component, and the relationships between them ▪ Classification of decision-makers, decision-making settings, type and scope of decisions, strengths and weaknesses, barriers and enablers ▪ Examples of decision-making criteria and types and sources of evaluation data used ▪ Interview and workshop protocols for ascertaining local decision-making systems and processes
SHARE 4: Exploring opportunities and methods for consumer engagement in resource allocation in a local healthcare setting	
▪ How can consumer and community values and preferences be systematically integrated into organisation-wide decision-making for resource allocation?	▪ Model for integrating consumer values and preferences into decision-making for resource allocation ▪ Definitions for consumer engagement terminology ▪ Examples of sources of consumer information and data ▪ Examples of consumer-related activities generating proactive decisions to drive change
SHARE 5: Developing a model for evidence-driven resource allocation in a local healthcare setting	
▪ What are the implications for disinvestment at Monash Health? ▪ What is the most appropriate and effective approach to organisation-wide, systematic, integrated, evidence-driven disinvestment at Monash Health? ▪ Can a model for evidence-driven resource allocation in the local healthcare setting be derived from the Monash Health program to enable replication and testing?	▪ Model for exploring Sustainability in Health care by Allocating Resources Effectively in the local healthcare setting ▪ Definition of four program components, aims and objectives, relationships between components, principles that underpin the program, implementation and evaluation plans, and preconditions for success and sustainability. ▪ Summary of implications for disinvestment in the local setting and resulting decisions for program development ▪ Summary of factors for program sustainability ▪ Evaluation framework and plan
SHARE 6: Investigating methods to identify, prioritise, implement and evaluate disinvestment projects in a local healthcare setting	
▪ What methods are available to identify potential disinvestment opportunities in a local health service? ▪ What methods are available for prioritisation and decision-making to initiate disinvestment projects in a local health service? ▪ What methods are available to develop, implement and evaluate disinvestment projects in a local health service? ▪ What were the processes and outcomes of application of these methods at Monash Health? ▪ What factors influenced the decisions, processes and outcomes?	▪ Framework for evaluation and explication of a disinvestment project ▪ Examples of criteria for selection of disinvestment projects ▪ Methods for developing an evidence-based catalogue of potential disinvestment opportunities ▪ Algorithm for selecting a disinvestment project from an evidence-based catalogue of potential disinvestment opportunities ▪ Summary of barriers and enablers to implementation and evaluation ▪ Summary of factors related to determinants of effectiveness arising in SHARE process and disinvestment projects
SHARE 7: Supporting staff in evidence-based decision-making, implementation and evaluation in a local healthcare setting	
▪ What is current practice in accessing and using evidence for making, implementing and evaluating decisions at Monash Health? ▪ What decisions were made and outcomes achieved in the piloting of support services? ▪ What factors influenced the decisions, processes and outcomes?	▪ Matrix of barriers, enablers, additional needs and evidence-based interventions mapped to their corresponding components in four support services to enable evidence-based decision-making, implementation and evaluation ▪ Summary of factors influencing decision-making for development of support services ▪ Summary of factors influencing the outcomes of the SHARE support services piloting process ▪ Summaries of current practice, knowledge, skills, confidence and needs in finding, accessing and using evidence for making, implementing and evaluating decisions; and preferred formats for education and training ▪ Summaries of nature, type and availability of local health service data; data sources; uses and expertise available ▪ Evaluation framework and plan
SHARE 8: Developing, implementing and evaluating an Evidence Dissemination Service in a local healthcare setting	
▪ What are the potential features of an Evidence Dissemination Service in a local healthcare setting? ▪ How can high quality synthesised evidence be identified, captured, classified, stored, repackaged and disseminated? ▪ How can disseminated evidence be used to enhance current practice and how can use of evidence be reported? ▪ What are the processes and outcomes of disseminating evidence to self-selected and targeted participants in a voluntary framework? ▪ What are the processes and outcomes of disseminating evidence to designated decision-makers in a mandatory governance framework? ▪ What factors influenced the decisions, processes and outcomes?	▪ Two models for an Evidence Dissemination Service (EDS) in a local healthcare service ▪ Methods for identification, capture, classification, storage, repackaging and dissemination of evidence ▪ Methods to facilitate use of disseminated evidence and reporting of outcomes ▪ Taxonomy for categorising publications ▪ Framework for evaluation and explication of implementation of health information products and services ▪ Summaries of factors influencing decisions, processes and outcomes in development and delivery of the EDS
SHARE 9: Conceptualising disinvestment in the local healthcare setting	
▪ Aims: To discuss the current literature on disinvestment from a conceptual perspective, consider the implications for local healthcare settings and propose a new definition and two potential approaches to disinvestment in this context to stimulate further research and discussion.	▪ Discussion of the disinvestment literature in relation to terminology and concepts, motivation and purpose, relationships with other health improvement paradigms, challenges, and implications for policy, practice and research in local healthcare settings
SHARE 10: Operationalising disinvestment in an evidence-based framework for resource allocation	
▪ Aims: To discuss the current literature on disinvestment from an operational perspective, combine it with the experiences of the SHARE Program, and propose a framework for disinvestment in the context of resource allocation in the local healthcare setting.	▪ Discussion of the disinvestment literature from an operational perspective in local healthcare settings ▪ Summary of theories, frameworks and models used in disinvestment-related activities ▪ Framework for evidence-based disinvestment in the context of resource allocation - Standardised definitions and concepts to underpin framework - Principles for resource allocation decision-making - Potential activities and settings for disinvestment - Potential prompts and triggers to initiate disinvestment decisions - Methods and tools for disinvestment - Barriers to disinvestment
SHARE 11: Reporting outcomes of an evidence-driven approach to disinvestment in a local healthcare setting	
▪ Aims: To consolidate the findings, discuss the contribution of the SHARE Program to the knowledge and understanding of disinvestment in the local healthcare setting, and consider the implications for policy, practice and research.	▪ Summary of outcomes of the SHARE Program ▪ Key messages ▪ Implications for research, policy and practice
SHARE National Workshop	
▪ Aim: To share knowledge of disinvestment and develop links for future collaborative work opportunities	▪ Summary of disinvestment activities from health policy, health economics and health service perspectives ▪ Tools for group activities discussing disinvestment concepts and decision-making ▪ Tools for individual activities to capture information about current practice and research in disinvestment ▪ Workshop presentations ▪ Workshop evaluation tool and findings ▪ Summary of key messages

### Outcomes and outputs

Outcomes are the changes that result from a program of activities. The outcomes of each investigation are reported and discussed in detail in the individual papers and summarised in the final paper [50].

Outputs are materials or methods produced in the delivery of a program that could be used to inform decision-making and planning for other programs, reproduced to save time and resources, or adjusted to suit local needs. The SHARE outputs may be useful resources for knowledge brokers, decision-makers and change agents in healthcare settings and offer opportunities for application, testing, refinement and theory development by researchers.

In addition to this suite of papers, the SHARE activities have also produced a range of outputs that includes summaries of concepts, definitions, current practice, needs, emerging issues, decision-making criteria and influencing factors; frameworks and models, a taxonomy and algorithm; sources of information and data; and survey instruments. These are collated in Table 1 and discussed below.

## Discussion

### Limitations

SHARE is a case study in a single public health service in the Australian health system which limits the generalisability to other contexts and settings.

It was developed as a health service improvement initiative, not a research project. However the importance of a research component was recognised at project inception and was built into the funding application and evaluation design [44, 64].

The project team responsible for delivering the SHARE Program at Monash Health were also the researchers investigating the processes undertaken. This has the potential to introduce subjectivity into the evaluations and limit insight if organisational assumptions are accepted without challenge. Extensive stakeholder involvement, transparency of methods and participation of an external evaluator in the role of ‘critical friend’ [64] were included in the SHARE processes to minimise these limitations.

Many of the findings are the first of their kind; while this provides more information than was previously available, it requires further confirmation or refutation in subsequent studies.

### Implications for policy and practice

#### Establishing a disinvestment program in a local healthcare setting

Several outputs from SHARE activities may assist others seeking to establish similar programs. The proposed organisation-wide framework for disinvestment brings together the definitions, concepts, principles, decision-making settings, and steps in the disinvestment process, and addresses barriers and enablers when it is possible to do so through systems change (Paper 10). It is broad and theoretical, but may be made more specific and practical in combination with the SHARE models for resource allocation in a local healthcare setting (Paper 5) and integrating consumer views and perspectives into the resource allocation process (Paper 4). Additional information that may be of use includes summaries of issues to consider in development of an organisational program for disinvestment (Paper 2); implications for disinvestment in the local setting (Paper 5); factors that influenced decisions, processes and outcomes in disinvestment projects (Paper 6) and establishing services to support EBP (Papers 7 and 8); key messages from the SHARE Program (Paper 11); and theories proposed or applied in disinvestment-related projects and frameworks, methods and tools developed by others (Paper 10).

#### Seeking local information

The SHARE Program undertook multiple surveys, interviews and workshops. The protocols and instruments developed may be suitable for replication or adaptation to meet the needs of other settings. The results are provided in summary in the papers and in detail in additional files, and are discussed in the context of the current literature. The topics include local implications of a disinvestment program (Paper 2); current practice, barriers and enablers to making, implementing and evaluating decisions for resource allocation (Paper 3); current practice, knowledge, skills, confidence, barriers, enablers and needs of decision-makers in finding, appraising and using evidence in decisions, implementation and evaluation (Papers 7 and 8); content and format of training programs and support services to facilitate EBP (Papers 7 and 8) and sources, content, utilisation, availability, access and reporting of local health service datasets (Paper 7).

#### Identifying opportunities and making decisions for disinvestment

At the commencement of the SHARE Program Monash Health leaders did not have a complete or agreed understanding of where, how and by whom organisational decisions for resource allocation were made, implemented or evaluated. There was also a lack of this level of detail in the literature. The outputs of the investigation into decision-making systems and processes for resource allocation at Monash Health are reported in Paper 3 and include a framework for the process of resource allocation; classification of decision-makers, decision-making settings, type and scope of decisions; details of strengths and weaknesses, barriers and enablers; and examples of decision-making criteria used in a local healthcare setting.

A separate investigation, specifically considering disinvestment, evaluated methods for identification, prioritisation and decision-making for disinvestment projects (Paper 6). Outputs from this project include an algorithm for selecting projects from a catalogue of TCPs that were demonstrated to be harmful or ineffective; examples of criteria for selection of disinvestment projects; a summary of barriers and enablers to implementation and evaluation; and a summary of factors influencing the process and outcomes of undertaking disinvestment projects within the SHARE Program.

#### Implementing and evaluating change initiatives

There is some discussion of implementation strategies in the disinvestment literature, however much of it is theoretical and the authors do not report application or evaluation of these strategies in the local health service context [49]. The need for evaluation of disinvestment projects is highlighted in the literature but little guidance is provided [49]. The SHARE papers provide practical information from actual experiences to guide others in similar situations. These include:

- ▪ summaries of barriers and enablers from SHARE activities related to implementing and evaluating health service decisions for resource allocation (Paper 3) and implementing a disinvestment project (Paper 6); and barriers and enablers to disinvestment as reported in the literature (Paper 10).
- ▪ summaries of influencing factors and strategies to address them (Papers 2, 5, 6, 7 and 8).
- ▪ completed checklists for success and sustainability, characteristics of interventions and/or determinants of effectiveness related to the overall SHARE Program (Papers 5 and 11), process of disinvestment (Paper 6) and establishment of services to support EBP (Papers 7 and 8).
- ▪ evaluation frameworks and plans related to the overall SHARE Program (Paper 5) and establishment of support services (Papers 7 and 8).
- ▪ a framework for evaluation of implementation of an evidence-based innovation was adapted for use in survey design to investigate decision-making processes for resource allocation (Paper 3) and evaluation design to map evaluation and research activities to the process of change (Paper 5), explore factors that influenced the processes and outcomes of identifying and undertaking disinvestment projects (Paper 6), and evaluate new health information products and services (Paper 8).

### Implications for research

The SHARE outputs are described above in the context of policy and practice. The same lists could be repeated for research where the specific products could be trialled and refined, tested in different contexts or used to develop new hypotheses.

The need for frameworks and models for disinvestment is widely acknowledged [26, 29, 30, 32, 34, 39, 68–72]. The SHARE Program has contributed three new conceptual frameworks and three models and adapted existing frameworks.

The frameworks include potential settings and methods to integrate disinvestment decisions into health service systems and processes (Paper 2), components of the resource allocation process (Paper 3), evaluation and explication of a disinvestment project (Paper 6), evaluation and explication of implementation of health information products and services (Paper 8), and organisation-wide disinvestment in the context of resource allocation (Paper 10).

The models include integrating consumer values and preferences into decision-making for resource allocation in a local healthcare setting (Paper 4), exploring sustainability in health care by allocating resources effectively in the local healthcare setting (Paper 5) and facilitating use of recently published synthesised evidence in organisational decision-making through an Evidence Dissemination Service (Paper 8).

The frameworks and models can be tested in clinical, management or policy contexts; for disinvestment, resource allocation or other decision-making processes. They are each based on multiple components and the relationships between them. A range of hypotheses could be developed for the components and their relationships which could be tested in a number of ways using various methodologies.

## Conclusions

This suite of projects extends the existing literature on disinvestment and addresses some of the notable gaps. The outputs may be as useful as the outcomes for those considering disinvestment in the policy, practice and research contexts.

## Abbreviations

CCE: Centre for clinical effectiveness
EBP: Evidence based practice
GDP: Gross domestic product
HTA: Health technology assessment
OECD: Organisation for Economic Co-operation and Development
SHARE: Sustainability in Health care by allocating resources effectively
TCPs: Technologies and clinical practices
